# A Phosphosite Mutant Approach on LRRK2 Links Phosphorylation and Dephosphorylation to Protective and Deleterious Markers, Respectively

**DOI:** 10.3390/cells11061018

**Published:** 2022-03-17

**Authors:** Antoine Marchand, Alessia Sarchione, Panagiotis S. Athanasopoulos, Hélène Bauderlique-Le Roy, Liesel Goveas, Romain Magnez, Matthieu Drouyer, Marco Emanuele, Franz Y. Ho, Maxime Liberelle, Patricia Melnyk, Nicolas Lebègue, Xavier Thuru, R. Jeremy Nichols, Elisa Greggio, Arjan Kortholt, Thierry Galli, Marie-Christine Chartier-Harlin, Jean-Marc Taymans

**Affiliations:** 1 University of Lille, Inserm, CHU Lille, U1172-LilNCog-Lille Neuroscience and Cognition, F-59000 Lille, France; antoine.marchand@inserm.fr (A.M.); alessia.sarchione@inserm.fr (A.S.); liesel-mary.goveas@inserm.fr (L.G.); mdrouyer@cmri.org.au (M.D.); memanuele@icloud.com (M.E.); maxime.liberelle@inserm.fr (M.L.); patricia.melnyk@univ-lille.fr (P.M.); nicolas.lebegue@univ-lille.fr (N.L.); 2 Department of Cell Biochemistry, University of Groningen, 9747 AG Groningen, The Netherlands; athanasopoulospanag@hotmail.com (P.S.A.); y.f.ho@rug.nl (F.Y.H.); a.kortholt@rug.nl (A.K.); 3 BioImaging Center Lille, UMS 2014—US 41—PLBS, F-59000 Lille, France; helene.bauderlique@ibl.cnrs.fr; 4 University of Lille, CNRS, Inserm, CHU Lille, UMR9020-UMR1277—Canther—Cancer Heterogeneity Plasticity and Resistance to Therapies, Platform of Integrative Chemical Biology, F-59000 Lille, France; romain.magnez@inserm.fr (R.M.); xavier.thuru@inserm.fr (X.T.); 5 Department of Pathology, Stanford University, Stanford, CA 94305, USA; rjnichols@stanford.edu; 6 Physiology, Genetics and Behavior Unit, Department of Biology, University of Padova, 35131 Padova, Italy; elisa.greggio@unipd.it; 7 Institute of Psychiatry and Neuroscience of Paris, Université Paris Cité, INSERM U1266, F-75014 Paris, France; thierry.galli@inserm.fr; 8 GHU-Paris Psychiatrie et Neurosciences, Hôpital Sainte Anne, F-75014 Paris, France

**Keywords:** LRRK2, phosphorylation, Parkinson’s disease, lysosome, RABs

## Abstract

The *Leucine Rich Repeat Kinase 2* (*LRRK2*) gene is a major genetic determinant of Parkinson’s disease (PD), encoding a homonymous multi-domain protein with two catalytic activities, GTPase and Kinase, involved in intracellular signaling and trafficking. LRRK2 is phosphorylated at multiple sites, including a cluster of autophosphorylation sites in the GTPase domain and a cluster of heterologous phosphorylation sites at residues 860 to 976. Phosphorylation at these latter sites is found to be modified in brains of PD patients, as well as for some disease mutant forms of LRRK2. The main aim of this study is to investigate the functional consequences of LRRK2 phosphorylation or dephosphorylation at LRRK2’s heterologous phosphorylation sites. To this end, we generated LRRK2 phosphorylation site mutants and studied how these affected LRRK2 catalytic activity, neurite outgrowth and lysosomal physiology in cellular models. We show that phosphorylation of RAB8a and RAB10 substrates are reduced with phosphomimicking forms of LRRK2, while RAB29 induced activation of LRRK2 kinase activity is enhanced for phosphodead forms of LRRK2. Considering the hypothesis that PD pathology is associated to increased LRRK2 kinase activity, our results suggest that for its heterologous phosphorylation sites LRRK2 phosphorylation correlates to healthy phenotypes and LRRK2 dephosphorylation correlates to phenotypes associated to the PD pathological processes.

## 1. Introduction

Parkinson’s disease (PD) is the most common neurodegenerative motor disease and the gene encoding the Leucine Rich Repeat Kinase 2 (LRRK2) protein is considered to be one of the most important genetic determinants in PD [1]. The importance of *LRRK2* in PD was first revealed from genetic linkage studies and several missense mutations have a confirmed link to PD [2,3,4] (reviewed in [5]). For instance, the gene mutations leading to the G2019S amino acid substitution gene is the most frequent and is found in Caucasians in 5% of autosomal dominant forms of the disease and in 1% to 40% of sporadic Parkinson’s patients according to the ethnic origin of patients [6]. In addition, genomic variations at the *LRRK2* locus are risk factors for sporadic forms of PD [7,8]. The neurodegeneration of PD is caused by environmental and genetic factors that contribute to accumulations of subtle cytotoxic effects leading to the death of dopaminergic neurons in the nigrostriatal system. In surviving neurons, accumulations of macromolecular aggregates rich in alpha-synuclein and lipids called Lewy bodies are observed [9,10,11]. Among other genetic factors associated with PD loci, at least 24 potential susceptibility genes are involved in the trafficking and regulation of endosomal/autophagic proteins, suggesting that there is a need to better understand the deficits of membrane trafficking in PD [12,13]. LRRK2 itself has also been implicated in membrane related processes such as autophagy, the endolysosomal pathway, extracellular vesicle release or synaptic transmission [14,15].

LRRK2 is found at endosomes, lysosomes and amphisomes in the brain of rats [16] and in vesicular structures of the human brain [17]. LRRK2 is reported to regulate different aspects of the lysosomal physiology (reviewed in [15]). For example, the LRRK2 mutant, G2019S causes an increase in the size and decrease in the pH of lysosomes in astrocytes [18]. LRRK2 G2019S also induces a reduction of lysosomal activities, through reduction of proteolytic activity assessed using a colorimetric and fluorescent detection named DQ-Red-BSA, as well as accumulation of alpha-synuclein inclusions. This phenomenon is reversed by LRRK2 kinase inhibition [15]. In primary neuron cultures expressing LRRK2 R1441C, pH of lysosomes is increased and activity of lysosomal enzymes decreased [19]. These cultures also show decreases in protein degradation and autophagosome-lysosome fusion events [20]. Maintaining a pH of 4.5–5 in the lysosomes is crucial for maintenance of lysosomal enzymes and to obtain optimal protein degradation [21]. Induced pluripotent stem cells (iPSCs) expressing LRRK2 G2019S and R1441C are reported to show) decreased activity of GBA (glucocerebrosidase), an enzyme localized in the lysosomes known as a risk factor for PD, through regulation of RAB10, a LRRK2 kinase substrate [22]. This process is reported to be related to the increase in mutant LRRK2 mediated phosphorylation of RAB10 at the LRRK2 dependent site T72 [23]. RAB10 overexpression increases the activity of glucocerebrosidase, but overexpression of phosphomimic RAB10 T72E had no effect on GBA activity [22]. Interestingly, LRRK2 is recruited to lysosomes in cell culture after treatment with lysosomotropic agents [24,25].

LRRK2 interacts with a large number of vesicular proteins including proteins regulating membrane fusion events such as Rabs and SNAREs [26,27]. Recent work shows that LRRK2 collaborates with RAB proteins to operate on endolysosomal homeostasis by regulation of the balance between membrane repair or degradation through lysophagy. LRRK2 recruits RAB8a to the lysosomal membrane in macrophages after lysosomal damage [28], and LRRK2, RAB8a and RAB10 are found to be enriched in chloroquine stressed lysosomes [25]. Another study reports that the partnership between LRRK2 and RAB8a/RAB10 mediates centrosomal and ciliogenesis deficits in several cell types including patient’s derived samples [29,30,31].

LRRK2 is a multidomain and multiphosphorylated protein. Its phosphorylation sites can be divided into two categories, autophosphorylation sites and heterologous phosphorylation sites. A heterologous phosphorylation cluster is found between the ankyrin repeat (ANK) domain and leucine-rich repeat (LRR) domain at Serines S860, S910, S935, S955, S973 and S976 [32,33]. Similarly, a cluster of autophosphorylation sites is observed at the level of the GTPase domain of LRRK2 (the Ras of complex(ROC) domain), as well as other sites such as S1292 in the LRR domain [34,35]. Interestingly, the level of phosphorylation at the ANK-LRR interdomain region is found to be decreased in brains tissues of Parkinson’s patients [36], while autophosphorylation levels (at S1292) are increased [37] in brains of Parkinson’s patients compared to controls.

LRRK2 binds 14-3-3 at LRRK2 phosphorylation sites of both the ANK-LRR interdomain region and ROC domain [38,39]. This binding is proposed to be regulated by the phosphorylation profile of LRRK2, including the S910 and S935 phosphorylation sites [40]. After kinase inhibition with type I kinase inhibitors, LRRK2 phosphorylation is decreased and the binding to 14-3-3 is disrupted. This loss is correlated with the relocalization of LRRK2 into skein-like structures [38,39]. LRRK2, when its localization is associated with membranes, presents greater kinase activity in comparison to cytosolic LRRK2 [41].

While these previous observations point to the importance of phosphorylation in normal LRRK2 functions, as well as in PD, the precise downstream effects of phosphorylation changes in LRRK2 remain poorly known. For these reasons, we aimed to study phenotypic consequences of LRRK2 phosphorylation on LRRK2′s biochemical and cell physiological properties. We used phosphorylation/dephosphorylation mimicking mutants at the ANK-LRR domain of LRRK2 and tested how these changes affect LRRK2′s catalytic activity, interaction and phosphorylation of vesicular partners, recruitment to discrete subcellular compartments and effects on neurite outgrowth.

## 2. Materials and Methods

### 2.1. Cell Culture

HEK293T cells (ATCC ref CRL-3216) were grown in Dulbecco’s modified Eagle’s medium containing high glucose 4500 mg/L (Thermo Fisher Scientific, Waltham, MA, USA) and 10% fetal bovine serum, HEPES 25 mM (Life Technologies, Carlsbad, CA, USA), Penicillin-Streptomycin 20 U/mL (Thermo Fisher Scientific, Waltham, MA, USA) at 37 °C.

SH-SY5Y cells (ATCC ref CRL-2266) were grown in 50% DMEM-F12 and 50% MEM (Thermo Fisher Scientific, Waltham, MA, USA) containing 10% fetal bovine serum, non-essential amino acids (Life Technologies, Carlsbad, CA, USA), L-Glutamine 1X (Life Technologies, Carlsbad, CA, USA), Penicillin-Streptomycin 20 U/mL (Thermo Fisher Scientific, Waltham, MA, USA) at 37 °C.

PC12 cells (ATCC ref CRL-1721) were cultured in RPMI 1640 medium (Thermo Fisher Scientific, Waltham, MA, USA) containing 10% fetal horse serum, 5% fetal bovine serum. Supplemented with Penicillin-Streptomycin 20 U/mL (Thermo Fisher Scientific, Waltham, MA, USA). Differentiated neuronal cells were grown on collagen-coated plates. Differentiation was induced with RPMI 1640 medium with 1% fetal horse serum and 0.5% fetal horse serum containing NGF 50 µg/mL, medium was changed every 48 H. Differentiation was completed when neurite network is fully developed, after 10 days of differentiation. In addition to normal PC12 cells, we used PC12 cells KO for VAMP2, VAMP4 and VAMP7 kindly provided by Dr. Thierry Galli (U1266, Institut de Psychiatrie et Neurosciences de Paris, Paris, France).

### 2.2. Plasmids and Stable Cell Lines

LRRK2 was cloned into the pLV-CSJ vector backbone which is itself constructed by replacing the mCherry sequence digested out with *BamH*I and *Nhe*I from pLV-mCherry (Addgene plasmid #36084) with a multiple cloning site via ligation of a short adaptor sequence containing the restriction digestion recognition sequences for *Bgl*II and *Nhe*I (New England Biolabs, Ipswich, MA, USA). The LRRK2 sequence cloned into the pLV-CSJ backbone were amplified from the p3flag-CMV10-LRRK2 plasmid (a generous gift from Prof. T. Iwatsubo, University of Tokyo, Japan) and a triple flag tag was introduced by adaptor ligation.

From this vector, mutations (S910A, S910D, S935A, S935D, S955A, S955D, S973A, S973D, S1292A, S1292D, S860/910/935/955/973/976A, S860/910/935/955/973/976D) were introduced using site directed mutagenesis. pLV-CSJ-mCherry-LRRK2 plasmids were generated by excising the 3flag sequence (restriction sites to include) and ligating the mCherry sequence (obtained by PCR using the pLV-mCherry and primers with sequences). All coding sequences were sequenced (Eurofins genomics) and digested with EcoRI and EcroRV enzymes (Thermo Fisher Scientific, Waltham, MA, USA). Lentiviral vectors (LV) encoding 3xFLAG-LRRK2 and mCherry-LRRK2 were prepared as previously described [42]. PC12 expressing pLV-CSJ-mCherry-LRRK2 were sorted with a FACSAria cell sorting flow cytometer (BD Biosciences, San Jose, CA, USA) to obtain a polyclonal culture of PC12 expressing LRRK2. The expression of 3xFlag-LRRK2 and mCherry-LRRK2 was validated by Western blotting.

### 2.3. Immunoblotting, Antibodies and Densitometry

Cells were lysed in lysis buffer (Tris 20 mM pH 7.4, NaCl 150 mM, EDTA 1 mM pH 8.8, Glycerol 10%, Triton x-100 1%, protease/phosphatase inhibitor 1X) for 30 mn on a rotating shaker at 4 °C followed by a clearing step by centrifugation at 15,000× *g* for 15 mn at 4 °C. Protein concentrations were measured using the BCA assay (Pierce Biotechnology, Rockford, IL, USA). Proteins samples were mixed with sample buffer (Thermo Fisher Scientific, Waltham, MA, USA) and boiled at 95 °C for 10 mn. Proteins were separated by 4–20% Tris-glycine SDS-PAGE and transferred on PVDF. Immunoblots were blocked in 5% nonfat dry milk in TNT 1X for 1 H and then incubated with primary antibodies overnight at 4 °C. Membranes were washed 3 times for 10 mn followed by 2 H incubation with secondary antibodies. Bands were visualized using enhanced chemiluminescence (Amersham, Buckinghamshire, UK) that was acquired using the image analyzer Imager 600 (GE Healthcare, Chicago, IL, USA) or using fluorescent secondary antibodies where signals were acquired using a Typhoon 9500 laser scanner (GE Healthcare, Chicago, IL, USA). Densitometric analysis of the bands on the blot images was performed using ImageQuant, Typhoon FLA 9500 or ImageJ software. The following primary antibodies were used: Mouse anti-LRRK2 (NeuroMab, USA, N241A/34, 1 µg/mL dilution), Rabbit anti-LRRK2 phospho S910 (Abcam; Cambridge, UK, UDD1, 1/1000 dilution), Rabbit anti-LRRK2 phospho S935 Abcam; Cambridge, UK, UDD2, 1/1000 dilution), Rabbit anti-LRRK2 phospho S955 (Abcam; Cambridge, UK, MJFR11, 1/1000 dilution), Rabbit anti-LRRK2 phospho S973 (Abcam; Cambridge, UK, MJFR12, 1/1000 dilution), Rabbit anti-LRRK2 phospho S1292 (Abcam; Cambridge, UK, MJFR19, 1/1000 dilution, note that for the quantification of signals from this antibody, the signal obtained with the LRRK2-S1292A phosphodead construct was used as background noise level that was subtracted from detection levels in the different experimental conditions), mouse anti-FLAG (Sigma-Aldrich, St. Louis, MO, USA, FLAG-M2, 1/1000 dilution), Rabbit anti-mCherry (Abcam; Cambridge, UK, 1/1000 dilution), Rabbit anti-Rab8a (Cell Signaling, Danvers, MA, USA, D22D8, 1:1000 dilution), Rabbit anti-Rab8A phospho T72 (Abcam; Cambridge, UK, MJF-R20, 1/1000 dilution), Rabbit anti-Rab10 (Cell Signaling, Danvers, MA, USA, D36C4, 1:1000 dilution), Rabbit anti- Rab10 phospho T73 (Abcam; Cambridge, UK, MJF-R21, 1/1000 dilution), Rabbit anti-Rab29 (Abcam; Cambridge, UK, MJF-R30-124, 1/1000 dilution), Rabbit anti-Rab29 phospho T71 (Abcam; Cambridge, UK, MJF-R24-17-1, 1/1000 dilution), Rabbit anti-EEA1 (Cell Signaling, Danvers, MA, USA, C45B10, 1:1000 dilution), Mouse anti-gamma tubulin (Abcam; Cambridge, UK, GTU-88, 1/10000 dilution), Mouse anti-LAMP2 (Abcam; Cambridge, UK, H4B4, 1/1000 dilution), Rabbit anti-LC3B (Abcam; Cambridge, UK, 1/1000 dilution).

### 2.4. LRRK2 Purification

Recombinant LRRK2 were purified as previously described [43,44]. In brief, HEK293T expressing 3xFLAG-LRRK2 were lysed in IP lysis buffer (Tris 20 mM pH 7.4, NaCl 150 mM, EDTA 1 mM pH 8.8, Glycerol 10%, Triton x-100 1%, protease/phosphatase inhibitor 1X) and incubated with anti-FLAG-M2-magnetic beads (Sigma-Aldrich, St. Louis, MO, USA) overnight at 4 °C. Beads were washed in different buffer (4X IP lysis buffer) followed by (2X elution buffer, Tris pH 7.4 25 mM, NaCl 200 mM, MgCl_2_ 5 mM, DTT 1 mM, Triton X-100 0.02%). LRRK2 proteins were eluted from the beads by competition with 0.1 µg/µL of 3xFLAG peptide in elution buffer for 30 mn at 4 °C on a vertical agitator. Note that for protein stability experiments, the same purification procedure is followed, using several alternate buffers to test LRRK2 stability in different buffers (cfr. infra). Quantities of purified protein were quantified by silver staining using standard dilution of BSA.

### 2.5. In Vitro Kinase Assay

The kinase activities of purified LRRK2 were measured at 30 °C in Kinase assay buffer consisting of Tris 25 mM (pH 7.5), MgCl_2_ 15 mM, β-glycerol phosphate 20 mM, sodium fluoride 1 mM, EGTA 1 mM, sodium orthovanadate 1 mM, DTT 2 mM and 0.1 mg/mL BSA. 0.1 µM purified LRRK2 and 75 µM LRRKtide peptides were used in the assay. The reaction was initiated by addition of adenosine triphosphate 25 µM, labeled on the gamma phosphate group with ^32^P (^32^P-γ-ATP) (4 Ci/mmol). At different time points until 30 min of reaction, the reaction mixtures were quenched in 100 mM ice-cold EDTA. The quenched samples were then spotted on P81 phosphocellulose paper discs pre-rinsed with 75 mM ice-cold phosphoric acid and further washed with phosphoric acid to remove free ATP. The paper discs were then air dried before scintillation counting.

A total of 40 ng of purified LRRK2 proteins were mixed with 200 ng of recombinant RAB8a (OriGene, Rockville, MD, USA) and 100 µM of ATP in kinase buffer (Tris pH 7.4 25 mM, MgCl_2_ 10 mM, DTT 2 mM, Triton X-100 0.02%) for 1 H at 37 °C. Phosphorylation of RAB8a was assay by SDS-PAGE.

### 2.6. GTPase Activity

The GTPase activities of purified LRRK2 were measured at 30 °C in GTPase assay buffer consisting of Tris 50 mM (pH 7.5), NaCl 150 mM, MgCl_2_ 10 mM, 5% Glycerol, β-mercaptoethanol 3 mM and 0.2 mg/mL BSA. 0.1 µM purified LRRK2 was added into 500 µM 32P-γ-GTP (0.15 Ci/mmol) to initiate the reaction. At different time points until 180 min of reaction, the reaction mixtures were quenched in 5% activated charcoal in 20 mM phosphoric acid. The quenched samples were then spun down by table-top centrifuge at 20,000× *g* for 10 min and the supernatants were aspirated for scintillation counting.

### 2.7. Protein Stability Assay and Microscale Thermophoresis Analysis

For assessment of stability of purified proteins, the purification buffer described above was used, using several combinations of alternate buffers, based on previous publications. Two lysis buffers were used: lysis buffer 1 (LB1)—20 mM Tris pH 7.5, 150 mM NaCl, 1 mM EDTA, 1% Triton, 10% Glycerol at pH 7.4 and pH 8 [45] and lysis buffer 2 (LB2) HEPES 20 mM pH 6.8, NH_4_CL 100 mM, MgCl_2_ 5 mM, CaCl_2_ 10 mM, Triton 1%, Glycerol 5% [44]. Wash steps were performed using wash buffer (Tris pH 7.5 25 mM, NaCl 400 mM, Triton 1%) at pH 7.4 or pH 8, or in lysis buffer LB2. Elutions were performed in the following elution buffers: Tris HCl 25 mM pH 7.4 or 8.0, NaCl 200 mM, MgCl_2_ 5 mM, DTT 1 mM, 0.02% Triton with 0.1 µg/mL 3xFlag peptide or in 20 Mm HEPES pH 8.2, NH_4_Cl 100 mM, MgCl_2_ 5 mM, CaCl_2_ 10 mM, 0.02% Triton with 0.1 µg/mL 3xFlag peptide. The freshly eluted proteins were then analyzed using a label free thermal shift assay in the Nanotemper TychoTM (NanoTemper Technologies, Munich, Germany).

For assessment of binding of LRRK2 with 14-3-3, we used the microscale thermophoresis method. After the expression of mCherry-LRRK2 in HEK293T, cells were lysed in lysis buffer 1 (described above) and 5 µM of recombinant 14-3-3zeta (Abcam, UK) is added to the lysate. The mix is then placed into Monolith NT.115 Premium Capillaries (NanoTemper Technologies, Munich, Germany) and analyzed using the Nanotemper Monolith Pico (NanoTemper Technologies, Munich, Germany) at 10% of power using the pico RED excitation color.

### 2.8. Immunocytochemistry

SH-SY5Y stably expressing 3xFLAG-LRRK2 WT were grown on coverslips coated with Cell tak solution (292.5 µL of bi-carbonate buffer, 2.5 µL of NaOH 1 M, 5 µL of Cell Tak). Cells were fixed using 4% paraformaldehyde for 15 mn at room temperature, followed by 3 PBS washes. Cells were permeabilized by PBS-Triton-X100 0.1% for 5 mn at room temperature. Cells were blocked for 20 mn with PBS-BSA 0.5% and incubated with primary antibody diluted at 1:1000 in PBS-BSA 0.5% overnight at 4 °C, washed 3 times with PBS and incubated for 2 h at room temperature with secondary antibody diluted at 1:1000 in PBS-BSA 0.5%. Coverslips were mounted in Prolong Gold Antifade mounting medium (Invitrogen, Montigny le Bretonneux, France). Pictures were taken on Zeiss LSM 710 confocal microscope (Zeiss, Oberkochen, Germany).

### 2.9. Lysosomal Isolation

HEK293T cells expressing 3xFLAG-LRRK2 were cultured for 24 h in a medium containing an iron-coupled dextran. This compound accumulates in lysosomes. After a washout period of 24 H in a medium without dextran the cells were centrifuged 60× *g* for 5 mn then washed with PBS. A mechanical lysis was performed with a Dounce Tight followed by 8 passes in a 23 G needle. After a 400× *g* centrifugation for 10 min, the lysate was placed on a magnetic column (MS Columns, Miltenyi Biotec, Paris, France). The lysosome-enriched fraction was then collected by detaching the column from the magnetic base in PBS/0.1 mM sucrose buffer. The samples were then analyzed by Western blot.

### 2.10. Image Stream Analysis

Five million PC12 cells are collected by centrifugation (500 g, 5 mn) and resuspended in 1 mL of medium containing 10 µg Hoechst 33258 (Invitrogen, Montigny le Bretonneux, France). After 30 mn incubation at 37 °C, cells were again centrifuged 500 G for 5 mn and incubated in medium containing 75 nM of Lysotracker Deep Red (Thermo Fisher Scientific, Waltham, MA, USA) and 75 µg of FDGlu (Tebu-Bio, Le Perray-en-Yvelines, France) for 1 h at room temperature. Finally, cells are centrifuged and resuspended in 20 µL of PBS + EDTA 0.5 M and analyzed using an ImageStream MKII (AMNIS) at 60X magnification at a low speed with a maximum laser power for 488 nm and 561 nm lasers (200 mW) and with 35 mW for 375 nm laser and 20 mW for 642 nm laser. Compensations were carried out using single stained controls. Around 50,000 cells were recorded in order to have sufficient a number of cells for statistical analysis. Then data were analyzed with IDEAS Software (Millipore, Billerica, MA, USA) using successive masks and features, as described in the Results section.

### 2.11. Incucyte Live Cell Analysis

To analyze the neurite complexity, the Incucyte^®®^ S3 live-cell imaging system (Sartorius, Minisart, Göttingen, Germany) was used. A total of 4000 PC12 cells stably expressing mCherry were seeded on collagen coated Costar^®®^ 24-well Clear TC-treated Multiple Well Plates (Corning, Corning, NY, USA). Differentiation was induced with RPMI 1640 medium with 1% fetal horse serum and 0.5% fetal bovine serum containing 50 µg/mL NGF. This medium was refreshed every 48 h until full differentiation at day 10. Plates were scanned using the Incucyte system using a 20× objective and 36 images were taken per well. Assessment of neurite length and branch points was performed using the Incucyte^®^ Neurotrack analysis software module (Sartorius, Minisart, Göttingen, Germany). Average neurite length and branch points per cell were calculated by dividing total neurite length or branch points per well by the cell confluence expressed in mm^2^.

### 2.12. Statistical Analysis

All data are presented as means ± SEM. N is the number of independent experimental replicates. Statistical significance was determined using a Mann–Whitney analysis for a comparison of two experimental conditions, or a Kruskal–Wallis test followed by a post hoc Dunn test for multiple comparisons or a 1-way ANOVA followed by a Dunnett’s test for multiple comparisons when more than 2 experimental conditions were compared. All statistical analyses were performed using GraphPad Prism version 9 (GraphPad Software, San Diego, CA, USA).

## 3. Results

### 3.1. LRRK2 Phosphorylation Is Affected by Its Phosphorylation Mutants

First, to determine if the phosphorylation motif of LRRK2 could induce new phosphorylation profile we mutated the Serine (S) residues of position 910, 935, 955, 973, and 1292 to Alanine (A) to mimic non-phosphorylated Serines and to Aspartate (D) to mimic phosphorylated Serine. A compound mutant was also generated to mimic phosphorylated/non-phosphorylated LRRK2 presenting six mutations of Serines to Alanines or Aspartates in the heterologous phosphorylation cluster noted 6xA or 6xD (mutation of Serines 860/910/935/955/973/976 to Alanines (6xA) or Aspartates (6xD)).

**Figure 1:**
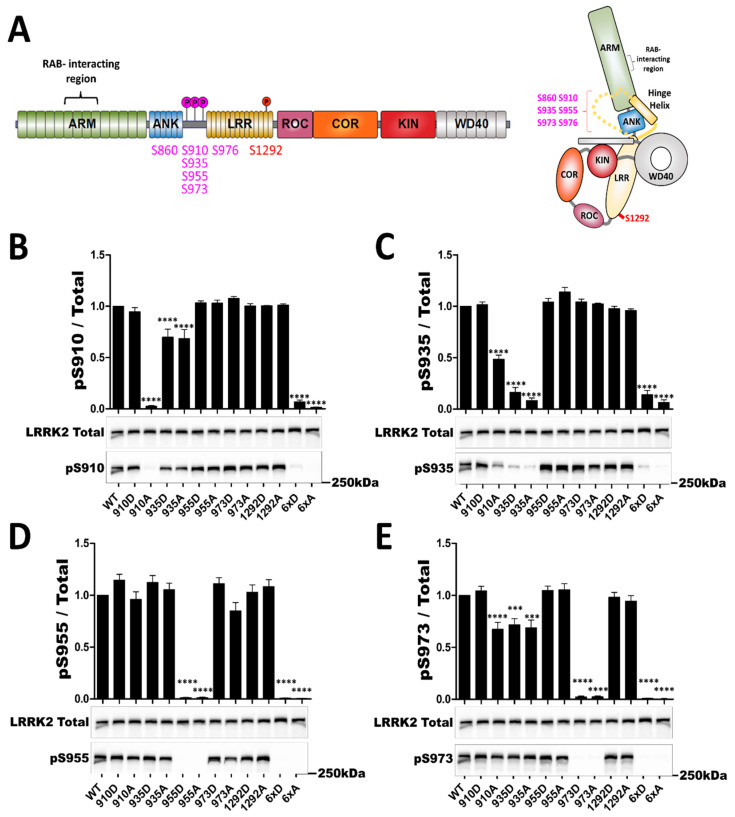
*In cellulo* phosphorylation profile of LRRK2 phosphosite mutants. Western blot analysis of HEK293T cell lysates after transfection of LRRK2 phosphomutants. LRRK2 protein domain representation with studied phosphorylated sites (heterologous sites are represented in purple and autophosphorylation site in red). (**A**) Schematic representation of LRRK2 domains, as well as the spatial arrangement of LRRK2 domains, indicating locations of phosphosites studied in the present work as well as the reported RABs interaction region. Unresolved portions of the structure are represented as dashes. Adapted from [46]. The ratio of phosphorylated site over total LRRK2 signal is shown in (**B**) for S910, (**C**) for S935, in (**D**) for S955 and in (**E**) for S973. The blot image for total LRRK2 is reused for illustration purposes in (**B**–**E**). The data represents the mean ± SEM from four independent experiments. The quantification of the signals was done by using Typhoon FLA 9500 and ImageQuant software (ImageQuant Tl (IQTL) 8.1, GE Healthcare, Chicago, IL, USA). The data were analyzed by One-way-ANOVA followed by a Dunnett’s test, using the WT condition as control. (*** *p* < 0.001), (**** *p* < 0.0001).

Phosphodead mutants S910A and S935A, but not S955A and S973A, changed the phosphorylation of neighboring Serines (Figure 1), matching previous observations [38,40]. By comparison, phosphomimicking mutants of these same sites presents the same effect on neighboring Serines as the phosphodead mutants, except S910D which does not display altered S935 phosphorylation. Of note, S910D is the only phospho-mimetic mutant to be recognized by the corresponding anti-phospho antibody, suggesting that the aspartate substitution in this position may effectively resemble phosphorylation.

We reported no significant effect on the cellular localization of LRRK2 mutants (Supplementary Figure S1). Of note, the phosphodeficient mutant LRRK2 6xA does not present skein-like structures in basal conditions. It is also noteworthy that we observe the presence of skein-like structure with MLi-2 treatment in all cases. Interestingly, mimicking the dephosphorylation of six sites in the ANK-LRRK domain does not result in a loss of binding with 14-3-3 in the experimental conditions described in materials and methods (Supplementary Figure S2).

### 3.2. Heterologous Phosphorylation of LRRK2 Affects Its Autophosphorylation

**Figure 2:**
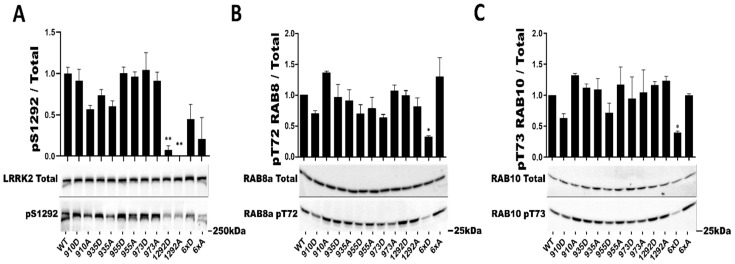
In cellulo LRRK2 kinase activity assays of LRRK2 phosphorylation mutants. Western blot analysis of HEK293T cell lysates after transfection of LRRK2 phosphomutants. The ratio of phosphorylated site over total protein signal is shown, (**A**) for LRRK2 autophosphorylation site S1292. Endogenous phosphorylation level of RAB8a T72 (**B**) and RAB10 T73 (**C**) were also quantified. The data represents the mean ± SEM from four independent experiments (**A**) and 3 independent experiments for (**B**,**C**). The quantification of the signals was done by using Typhoon FLA 9500 and ImageQuant software. The data were analyzed by One-way-ANOVA followed by Dunnett’s test using the WT condition as control. (* *p* < 0.05), (** *p* < 0.01).

Individually, S910 S935, S955, and S973 had no significant effect on the phosphorylation rate of the S1292 autophosphorylation site (Figure 2). In addition, while our mutants LRRK2 6xA and 6xD presented 30% lower average values for phosphorylation at S1292, these were likewise not statistically different compared to the WT control (Figure 2A). To assess a potential deficit in kinase activity of these two particular mutants, we measured in total cell lysate the phosphorylation of two known substrates of LRRK2, RAB8a and RAB10. Phosphorylation of both RAB8 T72 and RAB10 T73 was significantly decreased when LRRK2 6xD was expressed but in the case of LRRK2 6xA (Figure 2B,C).

**Figure 3:**
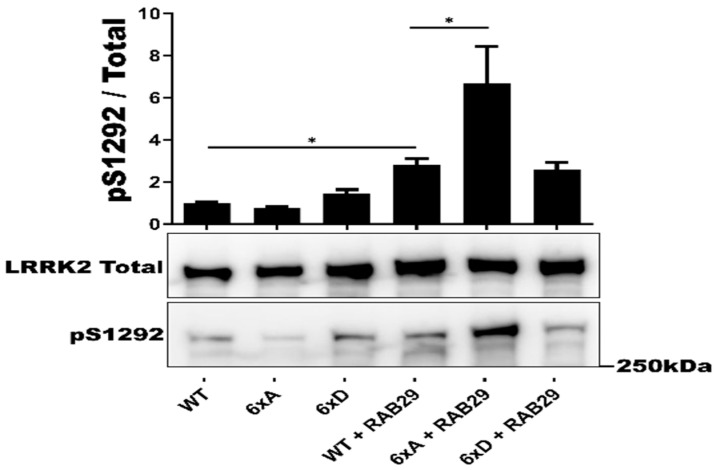
*In cellulo* LRRK2 activation by RAB29. Western blot analysis of HEK293T cell lysates after transfection of LRRK2 phosphomutants and 2myc-RAB29. The ratio of phosphorylated site over total protein signal is shown, LRRK2 autophosphorylation site S1292 was quantified. The data represent the mean ± SEM from 3 independent experiments. The quantification of the signals was done by using Imager 600 and ImageQuant software. The data were analyzed by One-way-ANOVA followed by a Tukey’s test. (* *p* < 0.05).

We then tested whether or not LRRK2 6xD could trigger dephosphorylation of RAB29 (Alias RAB7L1), a PD risk factor that has previously been reported as an interactor and substrate of LRRK2 [47,48]. Expression of the LRRK2 phosphorylation mutants 6xA and 6xD did not induce changes in RAB29-T72 phosphorylation compared to LRRK2 WR (Supplementary Figure S3). In this experiment, we decided to also test for effects of RAB29 on LRRK2, given previous reports that RAB29 overexpression activates autophosphorylation of LRRK2 at S1292 [49]. In this experiment, we found that expression of RAB29 led to an activation of LRRK2-S1292 phosphorylation of LRRK2 6xA that was 6-fold higher compared RAB29 induced activation of LRRK2-WT or LRRK2-6xD (Figure 3).

### 3.3. In Vitro Assay Is Not Affected by the Phosphorylation Profile of LRRK2

**Figure 4:**
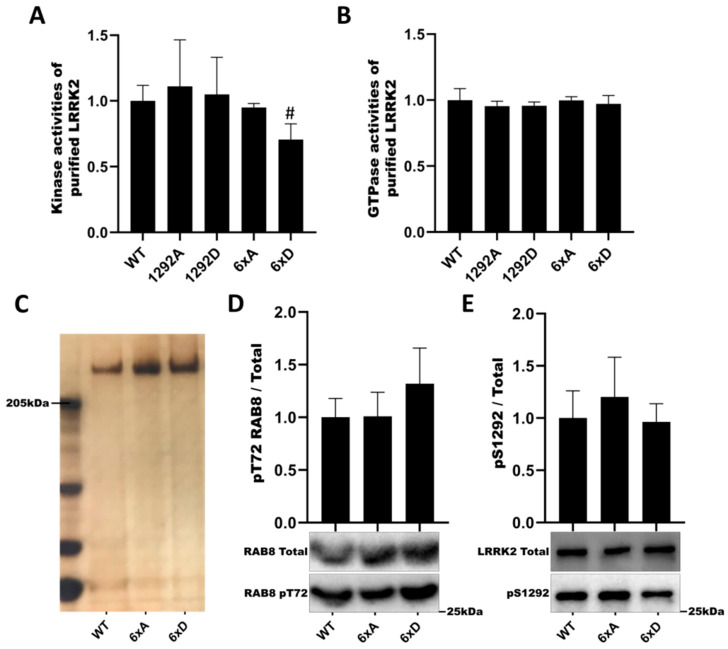
Catalytic activities of LRRK2 phosphorylation mutants. The kinase activities of purified LRRK2 were measured by addition of 32P-γ-ATP and LRRKtide for 30 mn (**A**) and the GTPase activities were measured independently by addition of 32P-γ-GTP for 180 mn (**B**) and then quenched. Samples were assay using scintillation counting. (**C**) Silver staining of purified LRRK2 proteins. (**D**) Purified proteins are incubated with recombinant RAB8a (Life technologies) and ATP for 60 min. Measurement of LRRK2 autophosphorylation level pS1292-LRRK2 (**D**) and level of RAB8a phosphorylation pT72-RAB8a (**E**). The data represent the mean ± SEM from three independent experiments and analyzed by Kruskal–Wallis test followed by a Dunn’s test using the WT condition as control. (# *p* = 0.0549).

Next, we assayed LRRK2 phosphomutants for in vitro kinase activity (LRRKtide peptide phosphorylation in the presence of ^32^P-γ-ATP) and GTPase activity (hydrolysis of ^32^P-γ-GTP). Mutants of phosphorylation site S1292 (S1292A, S1292D) had no effect on either activity (Figure 4A,B). However, in the multi-mutant LRRK2 6xD, kinase activity showed a trend to decrease (*p* = 0.0549) (Figure 4A). As we identified LRRK2 6xD to present *in cellulo* a decreased phosphorylation activity on RAB8a and RAB10, we tested LRRK2 multi-mutants for in vitro kinase assay on recombinant RAB8a. In addition, we verified the thermal stability of purified LRRK2 proteins and found that WT LRRK2 is of comparable stability to the LRRK2 6xA and LRRK2 6xD mutants (Supplementary Figure S4, while confirming a subtle improvement in thermal stability in the Hepes pH 8.2 buffer compared to the Tris buffers [44]. The losses of pT72-RAB8 and pS1292-LRRK2 phosphorylation observed *in cellulo* for the LRRK2-6xD mutant were not replicated in vitro and are not explained by changes in LRRK2 stability in vitro (Figure 4C–E).

### 3.4. NGF Induced Neurite Length and Branch Points in PC12 Cells Are Not Affected by Expression of LRRK2 WT or Phosphomutants

**Figure 5:**
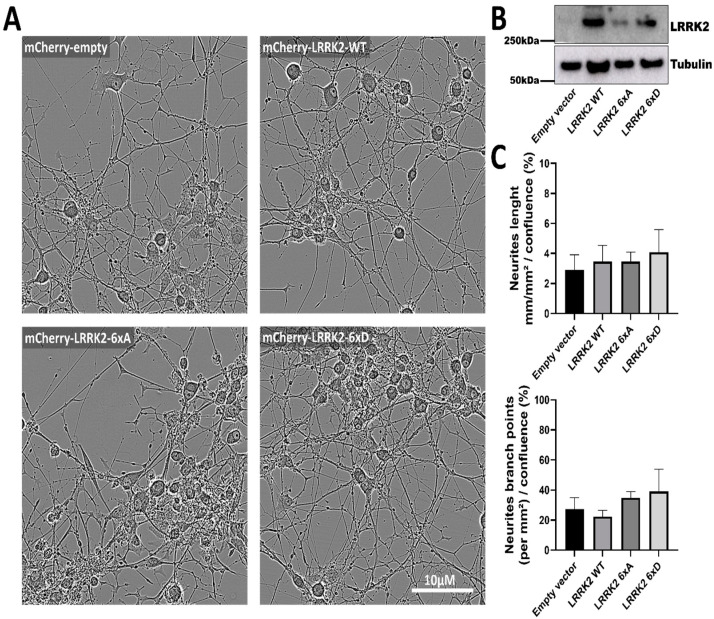
LRRK2 phosphorylation mutants do not affect NGF-induced neurite complexity in PC12 cells. PC12 transduced with mCherry-LRRK2 WT and phosphomutants are coated on P24 plate and differentiations are followed by live cell analysis (**A**). Expression of mCherry-LRRK2 was confirmed by Western blot analysis of cell lysates (**B**). Neurite length and neurite branch points were measured as described in the Materials and Methods (**C**).

LRRK2 mutants are reported to affect neurite length, for instance, LRRK2 mutant G2019S and R1441G cause neurite length reductions in primary cultured hippocampal neurons [50]. We generated PC12 cell line stably expressing mCherry-LRRK2 WT and compound phosphomutants to measure the neurite length of each construct, after 10 days of differentiation no change in neurite length or neurite branch points was shown between our phosphorylation mutants (Figure 5A–C).

### 3.5. LRRK2 and Its Phosphorylation Mutants Are Localized to the Lysosomes

**Figure 6:**
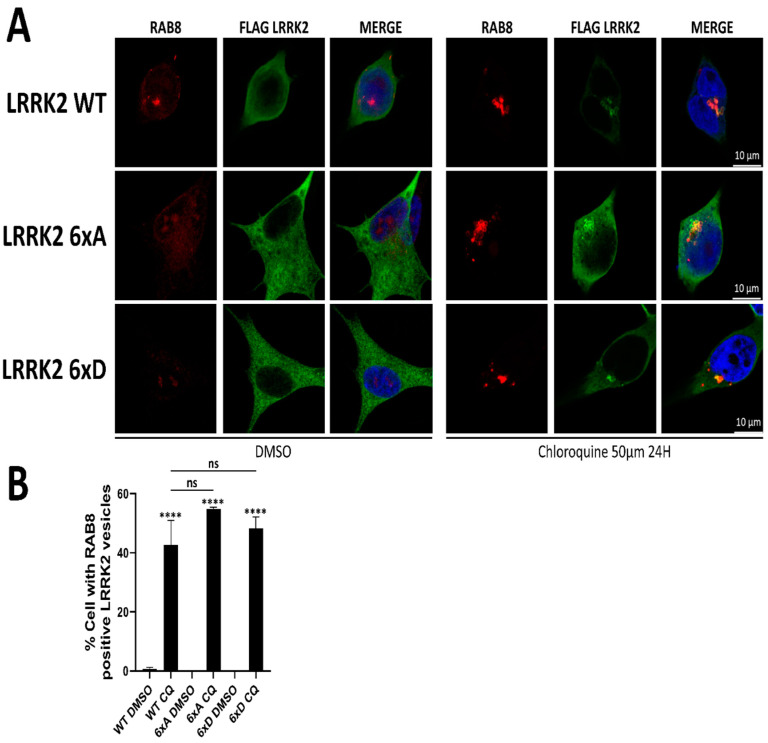
LRRK2 relocalizes to RAB8a positive structures after chloroquine treatment. (**A**) SH-SY5Y stably expressing LRRK2 were treated with 50 µM of chloroquine for 24 h prior fixation. (**B**) Quantification of the percentage of cells presenting RAB8a positive LRRK2 vesicles. Data are mean ± SEM (n = 3 independent experiments, 100–130 cells were analyzed per mutant in each experiment). The data were analyzed by One-way-ANOVA followed by Tukey’s test for multiple comparisons. (**** *p* < 0.0001, ns = non significant).

LRRK2 is reported to stabilize RAB8a and RAB10 on lysosomes when phosphorylated [25]. In addition, LRRK2 can relocalize to enlarged lysosomes to phosphorylate its substrates RAB8a and RAB10 [25]. To test if the reduced phosphorylation of RAB8a and RAB10 observed in cells above is due to a mislocalization of LRRK2 relative to RAB8a/RAB10 compartments, we tested colocalization of LRRK2 and RAB8a in SH-SY5Y stably expressing 3xFLAG-LRRK2 under basal conditions, as well as after chloroquine treatment, reported to recruit LRRK2 to RAB positive lysosomes [25]. While we confirmed the recruitment of LRRK2 to RAB8a positive compartments after choloroquine treatment, we found no changes in the subcellular distribution of LRRK2 6xA or 6xD compared to WT in any of the conditions tested (Figure 6B).

**Figure 7:**
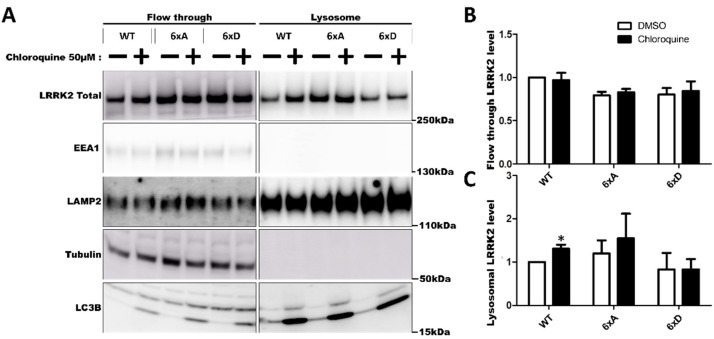
Abundance of LRRK2 in purified lysosomes. Lysosomes were magnetically isolated from HEK293T cells expressing 3xFLAG-LRRK2 and phosphorylation mutants as described in the Materials and Methods. Cells were treated with chloroquine 50 µM for 24 H to induce LRRK2 enrichment to lysosomes. Levels of LRRK2 in the flow through and in the lysosomes isolated are quantified by Western blotting (**A**). Relative abundance of LRRK2 in flow through (**B**) and in isolated lysosomes (**C**) is normalized with LAMP2 levels. The data represents the mean ± SEM from three independent experiments and are analyzed by one sample *t*-test (* *p* < 0.05).

**Figure 8:**
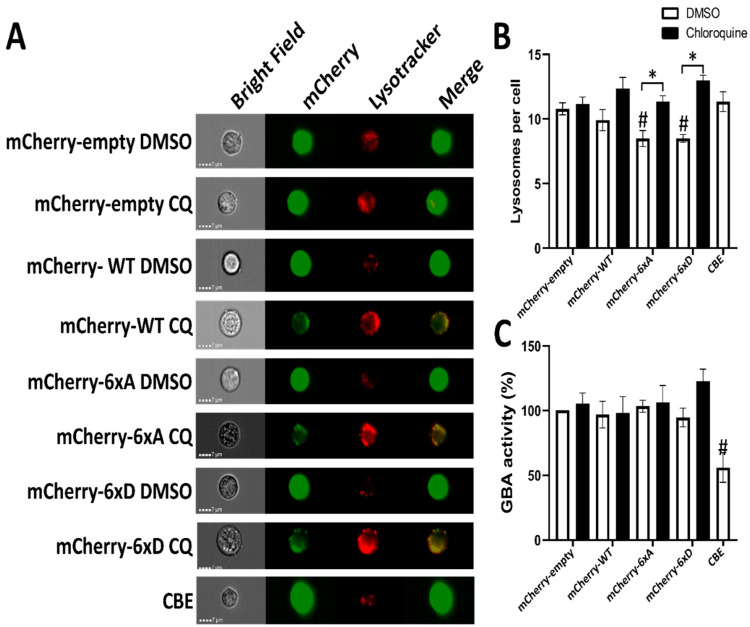
Lysosotracker analysis PC12 cells expressing WT and phosphomutant LRRK2 treated with and without chloroquine via imaging flow cytometry (ImageStream MKII, Amnis^®®^). PC12 cells were incubated with lysotracker for 1 h and subjected to ImageStream analysis. (**A**) Representative images of PC12 cells labeled with lysotracker, showing red colored positive spots corresponding to lysosomes. Green colored for LRRK2 staining. (**B**) Quantification of the number of spots imaged per cell. (**C**) Activity of GBA in each experimental condition is quantified. Data represent the mean spots detected from each condition. The data were analyzed by One-way-ANOVA followed by Dunnett’s test for multiple comparisons, using the mCherry-empty condition as control. (# *p* <0.05) or with one-sample *t*-test (* *p* < 0.05). (n = 4 independent experiments, 3000–5000 cells were analyzed per condition in each experiment).

To further investigate LRRK2 relocalization to the lysosomes, we measured abundance of LRRK2 in purified lysosomes (Figure 7). In our experiment, we were able to identify 3xFLAG-LRRK2 and its phosphorylation mutant in the isolated lysosomes (Figure 7C) without chloroquine treatment. Chloroquine treatment significantly increased the proportion of 3xFLAG-LRRK2 WT in the isolated lysosomes, but not for the conditions LRRK2-6xA and LRRK2-6xD (Figure 7C) while Rab8a phosphorylation remained low in the presence of LRRK2 6xD expression and chloroquine treatment (Supplementary Figure S5). In imaging flow cytometry, we were able to detect LRRK2 at the same position as lysotracker spots (Figure 8A).

To assess if LRRK2 phosphorylation influences the number of lysosomes in cells, we generated PC12 expressing LRRK2 and its 6xA/D mutants and control the number of lysosomes by imaging flow cytometry (ImageStream MKII, luminex). Chloroquine treatment did not increase the number of lysotracker spots in PC12 cells (Figure 8B). Expression of mCherry-LRRK2-6xA or 6xD reduced the number of lysotracker spot per cell and chloroquine treatment is able to restore the loss of lysotracker spots induced by expression of LRRK2 phosphomutants.

Lysosomal glucocerebrosidase activity in live PC12 cells was measured using the quenchable substrate FDGlu that allows us to measure in real time the activity of this enzyme [51]. In our experiments, LRRK2 overexpression or its phosphorylation had no effect on lysosomal glucocerebrosidase activity (Figure 8C).

## 4. Discussion

The question of the phenotypic consequences of LRRK2 phosphorylation or dephosphorylation has remained mostly unanswered [34,52,53]. Here, we present results that show that changes at the phosphosites in the ANK-LRR interdomain region affect LRRK2 activity in cells, including cellular kinase activity and RAB29 induced activation of LRRK2.

Using a phosphomutant approach, we first made a survey of LRRK2 phosphomutant variants, including modifications of individual sites compared to modifications of multiple sites. While some of the phosphodead mutants (Serine (S) to Alanine (A)) have been reported before, few reports have tested phosphomimicking mutants ((Serine (S) to Aspartate (D)) of LRRK2 and our report is also the first to include side by side comparisons of a broad range of phosphodead with phosphomimicking mutants. For those constructs that have been previously reported, such as the S910A or S935A mutants, our observations were similar to published observations, namely that these mutants affect the phosphorylation rate at other sites in the S910/S935 cluster, underscoring the robustness of our results [40,54]. The phosphomutant approach was thus implemented to verify which known phenotypes of LRRK2 are modified by LRRK2 phosphorylation.

Previous data indicates that several grey zones persist as to whether LRRK2 dephosphorylation is linked to its toxicity. LRRK2 is dephosphorylated in PD patient brains [36], and in cells with LRRK2 pathogenic mutations [34]. A special note can be made for LRRK2-G2019S, the most common LRRK2 mutation, where heterologous phosphorylation is unchanged in cell culture but reduced in brains, lungs and kidney of G2019S knockin mice [55,56,57]. Dephosphorylation also occurs in cells/tissues treated with LRRK2 type I inhibitors, that have also shown in several studies the ability to abolish adverse phenotypes induced by LRRK2 pathogenic mutations such as LRRK2 mutant induced neurite shortening [50]. Dephosphorylation of the N-TER region does not occur with Type II LRRK2 inhibitors, stabilizing LRRK2 in an open conformation [58]. Induction of filamentous accumulations of LRRK2, where LRRK2 binds to microtubules, is possible with LRRK2 stabilized in a closed conformation [58,59]. In our experiment, LRRK2 6xA and 6xD can also present filamentous cellular accumulations after treatment with MLi-2, a type I LRRK2 kinase inhibitor, suggesting that immobilization of LRRK2 in a closed conformation by MLi-2 is independent of the phosphorylation state of LRRK2 ANK-LRR domain. Interestingly, our previous work showed that cellular treatment with type I inhibitors of LRRK2 kinase leads to rapid recruitment of phosphatases to the LRRK2 complex (subunits of both protein phosphatase 1 and 2A holoenzymes), suggestive of a conformational change in LRRK2 [60]. We postulated that such a conformational change enables LRRK2 dephosphorylation, and given our observation that LRRK2 phosphomutants remain sensitive to type I inhibitor induced filamentous accumulations, we postulate that this phenomenon is related to a conformational change in LRRK2 rather than to dephosphorylation of LRRK2 *per se*.

Looking at cellular kinase activity, when we tested effects of the individual LRRK2 phosphosite mutants on phosphorylation of the LRRK2 substrates RAB8a and RAB10 in cells, we had no or low changes, while a significant decrease was observed for the 6xD mutant, consistent with a decreased kinase activity of LRRK2 in cells when it is phosphorylated. Intriguingly, when tested in vitro this result was not as pronounced as the *in cellulo* experiment, despite the observation that thermal stability of purified LRRK2 is not affected by phosphosite mutations. It is possible that, *in cellulo*¸ yet unidentified partners act as a sensor for the phosphorylation motif at the ANK-LRR and affects phosphorylation of its substrates in cells. We hypothesized that the decreased dephosphorylation of RAB8a and S1292 preferentially occurs when LRRK2 6xD is found in a cellular context, perhaps via the aid of additional proteins as a part of a LRRK2 complex.

While we showed *in cellulo* that LRRK2′s phosphorylation profile specifically regulates the phosphorylation of RAB8a and RAB10, we did not detect any effect on RAB29 phosphorylation. It has been reported that RAB29 binds to LRRK2 via the ankyrin domain and increased RAB29 expression is a trigger to activate LRRK2 kinase activity [49]. While the exact mechanisms of RAB29 induced activation of LRRK2 remains to be elucidated, our observation that the phosphodead variant of LRRK2 is significantly more activated than the phosphomimicking variant of LRRK2 suggests that LRRK2 phosphorylation is a modulator of LRRK2 activation. Further work would be warranted to explore the link between RAB29 mediated activation of LRRK2 and LRRK2 phosphorylation. For instance, a recent study has modeled binding between RAB38 and ARM domain, using a high resolution structure of LRRK2 [46], a similar analysis could be performed for LRRK2 and RAB29. Additionally, RAB29 overexpression recruits LRRK2 to the trans Golgi network and this is assumed to modulate kinases/phosphatases and stimulate LRRK2′s activity [49].

Another phenotypic effect of LRRK2 mutations is its ability to affect neurite outgrowth; however, the role of LRRK2 phosphorylation in this phenotype is not known. LRRK2 G2019S is reported to reduce stable contact between growth-cones [61] and G2019S impair neurite outgrowth in mice [62]. LRRK2 G2019S also increases the phosphorylation of RAB8a/RAB10 that are crucial factors required in rat hippocampal neurons to control neurite outgrowth [63,64]. In brief, a protein from the trans Golgi network, TRIO, regulates the axonal guidance by interacting with RABIN8, a guanine exchange factor (GEF) that activates RAB8/RAB10 [65]. RAB8a interaction with RABIN8 is dependent of the phosphorylation of RAB8a’s S111 phosphorylation site [66] the Rab8-RABIN8 interaction is also blocked with RAB8a T72 phosphorylation [67] and this interaction promotes neurite outgrowth [68]. Despite the reduced RAB8a and RAB10 phosphorylation in the presence of LRRK2-6xD, no significant effect on neurite complexity and length was detected in the tested conditions of NGF induced neurite outgrowth in PC12 cells. Our results suggest that other mechanisms may be at play besides Rab phosphorylation in LRRK2-mediated regulation of neurite complexity and further work is warranted, such as testing different experimental conditions (for instance testing other neurite forming cell types) and/or analyzing finer phenotypes, such as growth-cone formation deficits.

LRRK2 kinase activity has been associated with the regulation of lysosomal properties. Enhanced kinase activity results in enlarged lysosomes and reduced GBA activity and reduced lysosomal degradative properties [18,22,69,70]. Our results show that LRRK2 phosphorylation at S860/910/935/955/973/976 is not associated with modification of glucocerebrosidase activity. Interestingly, our results suggest that this cluster of Serine residues is involved in determining cellular lysosome numbers, as both the phosphodead and the phosphomimicking variants showed reduced numbers on lysosomes in PC12 cells (Figure 8B). Further work is needed to investigate the link between phosphorylation change and lysosomal dysfunction. Our results also suggest that the recruitment of LRRK2 to damaged lysosomes is not dependent on its ANK-LRR phosphorylation status. In the study by Eguchi et al. [25], LRRK2, in absence of chloroquine treatment, was not detected in the lysosomal compartment after lysosomal capture with iron-dextran beads, while our results show that we detect LRRK2 with and without chloroquine treatment in lysosomes. LRRK2 6xD abundance in lysosomes is not statistically different from the WT condition, with or without chloroquine treatment. The difference with the previous finding by Eguchi and colleagues could be explained by a higher expression level of LRRK2 resulting in a bigger proportion of LRRK2 in the lysosomes at the basal level. It is important to correlate these results with microscopy imaging where, without chloroquine, LRRK2 and RAB8a were not present in the same vesicular structures. Therefore, we conclude that LRRK2 can be recruited to lysosomes independently of its phosphorylation status, in the conditions tested.

Reduced phosphorylation of RAB8a and RAB10 observed in the presence of LRRK2 6xD phosphomimicking could be accompanied by reduced RAB35 phosphorylation, however this would need to be verified. Recent resolution of LRRK2 structure in its inactive state indicate that RAB proteins may bind to the ARM domain of LRRK2 [46]. In its inactive conformation, the ARM is remotely positioned relative to the kinase domain that is also covered by the LRR domain that wraps around the ATP binding cleft and block its access. The coordination of the RAB with the ARM domain suggests that LRR and ARM would shift positions in the active state to allow access of the RAB to the kinase.

## 5. Conclusions

Considering the postulates that enhanced kinase activity and increase of RABs phosphorylation are detrimental processes, our study results suggest that the activity of the phosphorylation mutant 6xD of LRRK2 at the ANK-LRR phosphosites corresponds to a protective state for LRRK2 while dephosphorylation mutant 6xA at these sites correspond to a deleterious state. Future research should pursue the investigations on this hypothesis to confirm a specific deleterious profile of phosphorylation, notably to understand the precise role of LRRK2 phosphorylation on disease related processes, such as LRRK2 effects on cytoskeleton, endolysosomal dysfunction, or cell viability.

## Supplementary Materials

The following supporting information can be downloaded at: https://www.mdpi.com/article/10.3390/cells11061018/s1, Figure S1: Qualitative analysis of the subcellular distribution of *LRRK2*, Figure S2: Assessment of interaction between LRRK2 and 14-3-3, Figure S3: In cellulo Rab29 phosphorylation by LRRK2, Figure S4: Protein stability of LRRK2 phosphorylation mutants. Figure S5: RAB8 phosphorylation in the presence of expression of LRRK2 WT or phosphomutant LRRK2 with our without chloroquine treatment.
