# Supplementary material for: A Phosphosite Mutant Approach on LRRK2 Links Phosphorylation and Dephosphorylation to Protective and Deleterious Markers, Respectively
Source: Cells. 2022 Mar 17;11(6):1018. doi: 10.3390/cells11061018 (PMC8946913; doi:10.3390/cells11061018)
Supplement: Supplementary file 1 [file cells-11-01018-s001.zip › cells-1540727-supplementary.pdf]

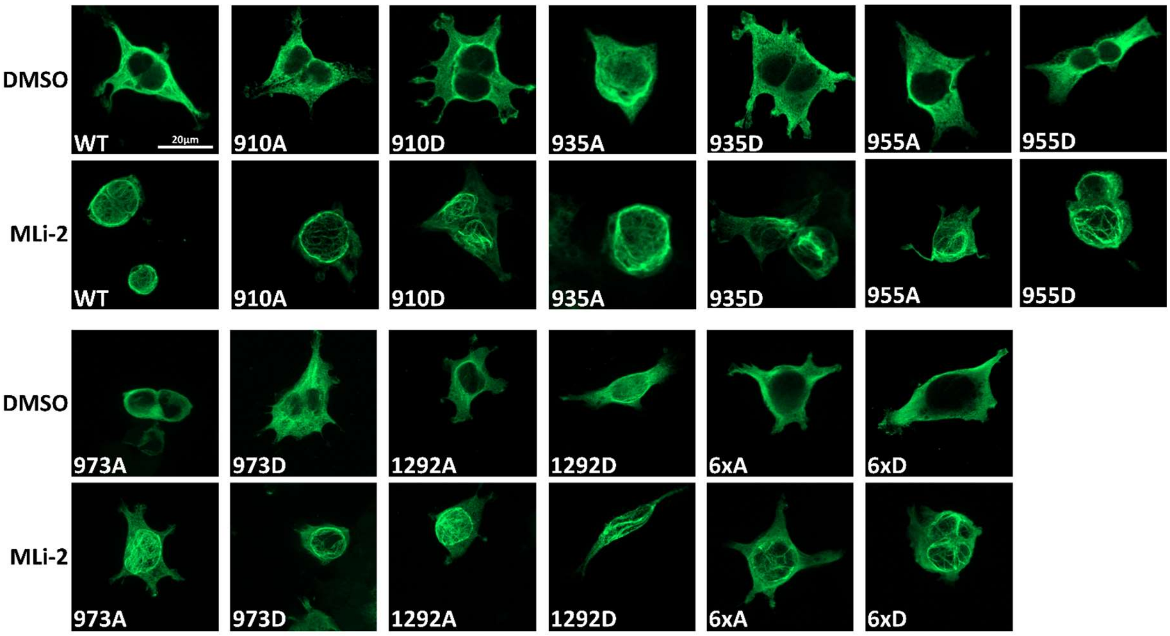

Supplemental Figure S1. Qualitative analysis of the subcellular distribution of *LRRK2*. Fluorescent microscope imaging of HEK293T expressing the indicated 3xFLAG-LRRK2 phosphorylation mutants are shown. Each phosphorylation mutant has been treated for 2 h with 10nM of the LRRK2 kinase inhibitor MLi-2 or the solvent DMSO. Scale bar, 20 µm.

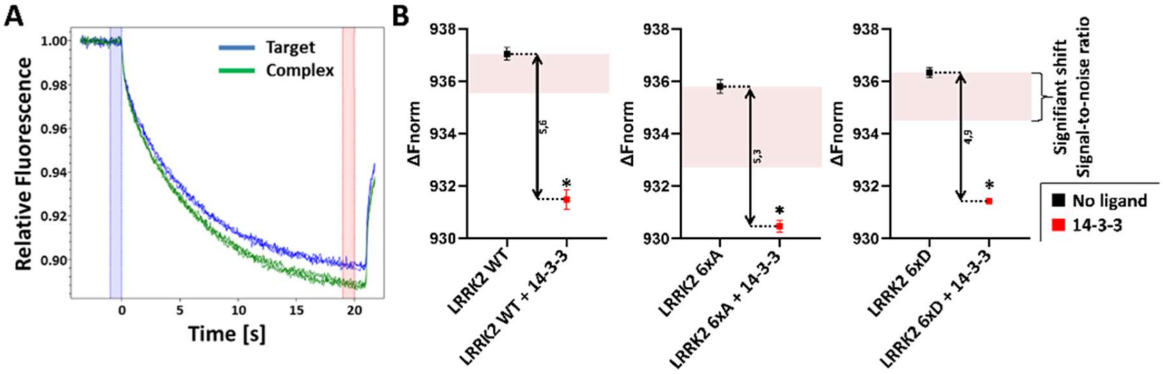

Supplemental Figure S2. *Assessment of interaction between LRRK2 and 14-3-3.* (A) MST raw data showing shift between after incubation of mCherry-LRRK2 + 14-3-3. (B) Binding check comparison between LRRK2 WT/6xA and 6xD with and without ligand. Significant shift signal noise-to-noise ratio is calculated by: Noise = 3x S/N, with S/N $= \frac{\text{Response amplitude}}{\sqrt{\frac{\sum_i (r_i - \bar{r})^2}{n-1}}}$.

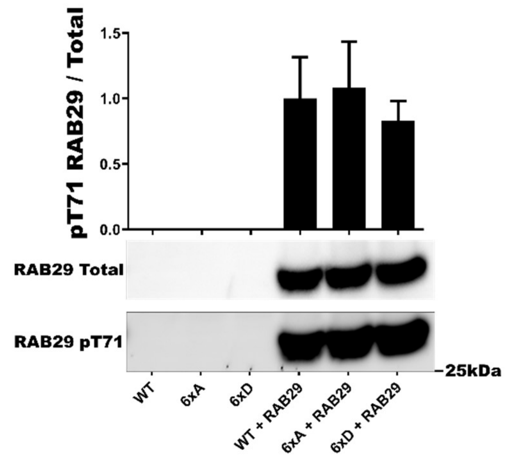

Supplemental Figure S3. In cellulo Rab29 phosphorylation by LRRK2. Western blot analysis of HEK293T cell lysates after transfection of LRRK2 phosphomutants and 2myc-Rab29. The ratio of phosphorylated site over total protein signal is shown, phosphorylation level of RAB29 T72. The data represents the mean ± SEM from four independent experiments. The quantification of the signals was done by using Imager 600 and ImageQuant software. The data were analyzed by One-way-ANOVA with Dunnett's multiple comparison. (* P <0.05).

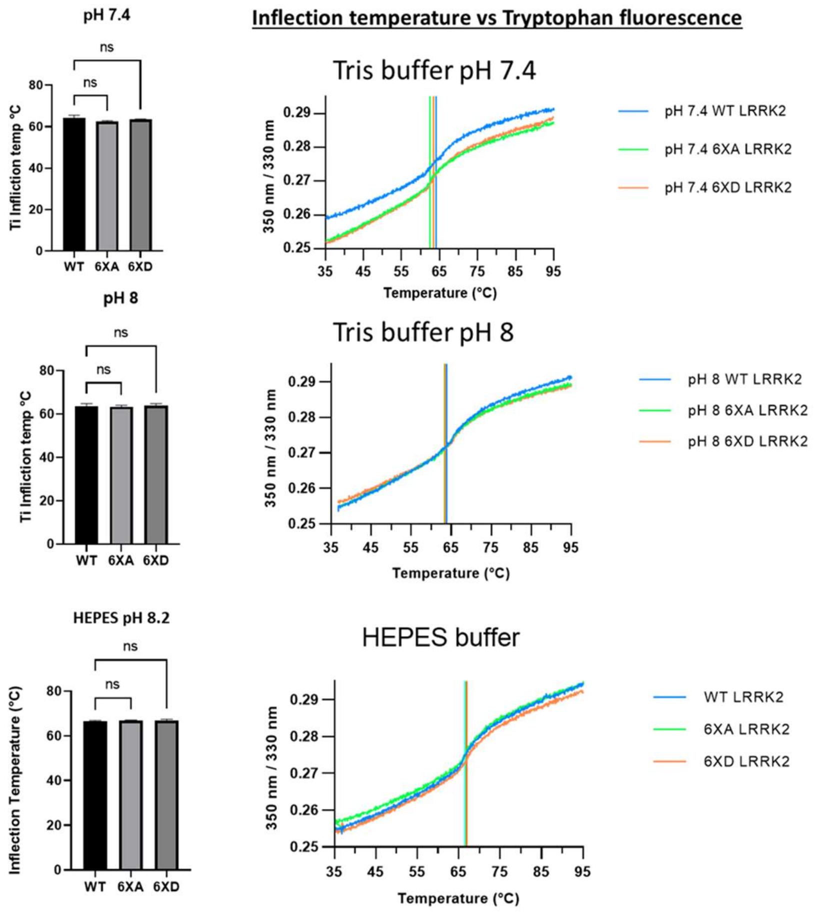

Supplemental figure S4. Protein stability of LRRK2 phosphorylation mutants. Thermal unfolding was measured by nano differential scanning fluorimetry using Nanotemper™ Tycho NT.6 (NanoTemper Technologies, Munich, Germany). Samples were loaded into Tycho NT.6 Capillaries (NanoTemper Technologies, Cat# TY-C001) and thermal unfolding profiles of LRRK2 WT and LRKK2 mutants were recorded. Temperature inflection values (Ti) were

obtained by automated data analysis. Protein unfolding was followed by tryptophan fluorescence intensity at 350 nm/330 nm. Temperature inflection (Ti) is the transition point along the curve which represents an unfolding transition of the sample. For determination of the protein inflection point where half of the protein is unfolded, either the fluorescence change in one of the two channels, or alternatively, the ratio of the fluorescence intensities (F350/F330) can be plotted.

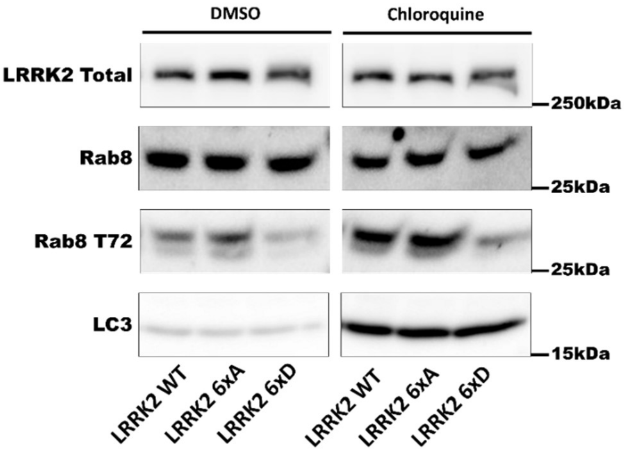

Supplemental figure S5. RAB8 phosphorylation in the presence of expression of LRRK2 WT or phosphomutant LRRK2 with our without chloroquine treatment. HEK293T were transfected with 3xFLAG-LRRK2 constructs and treated with chloroquine (50µM for 24H) 48 h post transfection. Lysates were analyzed by Western blot with the indicated antibodies. Reduced RAB8a phosphorylation with LRRK2 6xD overexpression in cells previously observed under basal conditions (see Figure 2) is also observed under chloroquine treatment.
